# Synthesis and Characterization of Rh/B–TNTs as a Recyclable Catalyst for Hydroformylation of Olefin Containing –CN Functional Group

**DOI:** 10.3390/nano8100755

**Published:** 2018-09-25

**Authors:** Penghe Su, Xiaotong Liu, Ya Chen, Hongchi Liu, Baolin Zhu, Shoumin Zhang, Weiping Huang

**Affiliations:** 1 College of Chemistry, Nankai University, Tianjin 300071, China; sph_edu@163.com (P.S.); jenalia@163.com (X.L.); xiaoyayaking@163.com (Y.C.); h335liu@edu.uwaterloo.ca (H.L.); zhubaolin@nankai.edu.cn (B.Z.); zhangsm@nankai.edu.cn (S.Z.); 2 The Key Laboratory of Advanced Energy Materials Chemistry (Ministry of Education), Nankai University, Tianjin 300071, China; 3 Collaborative Innovation Center of Chemical Science and Engineering, Tianjin 300071, China

**Keywords:** B-doped, Rh, TiO_2_ nanotube, hydroformylation, 2-methyl-3-butennitrile, functionalized olefin

## Abstract

The TiO_2_-based nanotubes (TNTs, B–TNTs) of different surface acidities and their supported Rh catalysts were designed and synthesized. The catalysts were characterized by X-ray diffraction (XRD), scanning electron microscopy (SEM), transmission electron microscopy (TEM), X-ray photoelectron spectrometer (XPS), tempera–ture–programmed desorption of ammonia (NH_3_–TPD), atomic emission spectrometer (ICP), and Brunauer–Emmett–Tellerv (BET) surface-area analyzers. Images of SEM and TEM showed that the boron-decorated TiO_2_ nanotubes (B–TNTs) had a perfect multiwalled tubular structure; their length was up to hundreds of nanometers and inner diameter was about 7 nm. The results of NH_3_-TPD analyses showed that B–TNTs had a stronger acid site compared with TNTs. For Rh/TNTs and Rh/B–TNTs, Rh nanoparticles highly dispersed on B–TNTs were about 2.79 nm in average diameter and much smaller than those on TNTs, which were about 4.94 nm. The catalytic performances of catalysts for the hydroformylation of 2-methyl-3-butennitrile (2M3BN) were also evaluated, and results showed that the existence of B in Rh/B–TNTs had a great influence on the catalytic performance of the catalysts. The Rh/B–TNTs displayed higher catalytic activity, selectivity for aldehydes, and stability than the Rh/TNTs.

## 1. Introduction

Hydroformylation of olefins is one of the most important homogeneous catalytic reactions in the chemical industry with a worldwide oxoaldehyde production [1]. By hydroformylation, one more carbon aldehyde than the original olefin can be obtained. Aldehydes are an important chemical raw material and act as intermediates in the synthesis of drugs, pesticides, natural products, and so on [2,3,4]. According to statistics, the production of aldehydes globally is now over 6.0 × 10^6^ t/a [5].

The typical hydroformylation of olefins is mainly catalyzed by homogeneous catalysis. Although the homogeneous catalyst system for hydroformylation has high catalytic activity, good selectivity, and other advantages, the transition metal complex used in the catalyst system may dissolve in the product, resulting in difficulties in the recovery of the catalyst [6,7]. Thus, there are two technical and scientific issues; one is the separation of the homogeneous catalyst from the product, and the other is preventing active components from loss. To resolve these issues, researchers have done much research on the effective separation of homogeneous catalysts and refraining from the loss of active components for the hydroformylation of olefins. One effective method is to “heterogenize” homogeneous catalysts. That is, the homogeneous catalyst, or the metal nanoparticles catalyst, is riveted on the materials of a large surface area [8,9,10,11,12,13,14]. Compared with conventional inorganic supports, the TiO_2_ nanotubes (TNTs) with multi-hydroxyls on the inner and outer surface can support the nanosized catalytic activity center, e.g., Rh nanoparticles. Furthermore, the multiwall of nanotubes may also act as the partition to separate Rh nanoparticles and prevent them from agglomeration. In addition, the nano-confinement effect of TNTs may have a marked impact on the selectivity of hydroformylation. In our previous studies, we used TiO_2_ nanotubes-supported Rh nanoparticles and amorphous Co–B catalysts to catalyze the hydroformylations of vinyl acetate [15] and cyclohexene [16]. According to previous reports, the acidity of the supports has a great influence on the catalytic activity. Olefin can be easily adsorbed by the Lewis acid positions [17,18], which is beneficial for the hydroformylation of olefin. The addition of B in the catalyst should be able to improve the catalytic activity of catalysts by increasing the Lewis acid positions [19,20]. Then, we used boron-modified TiO_2_ nanotubes-supported Rh-nanoparticle catalysts to catalyze the hydroformylations of styrene, and the TOF of aldehydes was up to 18,458 h^−1^ [21].

The hydroformylation of functionalized alkenes is an interesting topic [22]. The functional group (FG) in functionalized alkenes (C=C–FG), in which the FG is adjacent to the C=C group, may affect catalytic hydroformylation of C=C–FG by the chelation effect of FG with the active sites of the catalyst, which may lead to a decrease in the catalytic activity of the catalyst and the object control of regioselectivity of the catalytic hydroformylation of C=C–FG becomes very difficult. To reduce the impact of FG, we reported TNTs-supported Rh-Ru particle catalysts and compared their catalytic performances in the hydroformylation of vinyl acetate and cyclohexene [23]. The catalysts showed higher catalytic activities in the hydroformylation of vinyl acetate than that of cyclohexene because in the reaction, the main active site, Rh, can catalyze the main reaction efficiently, and the second active site, Ru, reduces the influence of the FG. Olefins containing the –CN group are important functionalized alkenes. Scientists have tried hard to regulate and control the regioselectivity of the catalytic hydroformylation of olefins containing the –CN functional group [24,25,26]. It is well-known that the control addition of two HCN to a single butadiene is still the most effective industrial process to synthesize adiponitrile today. When one molecule of HCN is added to a butadiene, the main byproduct is 2M3BN. 2M3BN, as is commonly known, can be used for the preparation of adiponitrile after isomerization, but investigating the hydroformylation of 2M3BN is an industrially important and scientifically challenging research subject. In this contribution, we report the design and synthesis of Rh-catalysts supported by TNTs of different surface acidity. The catalytic performances of catalysts synthesized for the hydroformylation of 2M3BN are investigated.

## 2. Materials and Methods

Butyl titanate, ethanol, H_3_BO_3_, NaOH, and nitric acid are analytical grade reagents and were purchased from commercial suppliers (Tianjin Guangfu Fine Chemical Research Institute, Tianjin, China) without any further purification. Deionized water is used in the experiments.

### 2.1. Preparation of the Catalysts

The preparation method of the TNTs was the same as our previous studies [27].

The preparation method of the B–TNTs is described as follows. Firstly, 15 mL butyl titanate was added into 225 mL absolute ethanol under constant stirring, marked as A. 1.01 g H_3_BO_3_ was dissolved in 15 mL distilled water respectively, and then the solution was slowly added to 225 mL absolute ethanol, marked as B. Solution A was mixed with solution B, and the pH was adjusted to 3 by using concentrated nitric acid, forming titanium sol containing 5 wt.% boron. After the titanium sols were aged for 1 day, it was dried at 80 °C and turned into titanium gel containing boron. Then, the gel was calcined for 2 h at 400 °C in a muffle furnace, and the boron-doped titanium dioxide powder was obtained. Thereafter, the as-prepared 1.0 g boron-doped titanium dioxide powder was treated with 60 mL 10 M NaOH aqueous solution in an autoclave for 12 h at 150 °C. The resulting material was washed with distilled water for it to become neutral and then dried at 60 °C, whereby a resulting white B–TNTs powder was obtained. The titanium sols containing 10 wt.% boron can be prepared by changing the amount of H_3_BO_3_ to 2.02 g. The actual B contents of the two B–TNTs powders synthesized and labeled as B–TNTs (b) and B–TNTs (c) were 0.56 and 0.99 wt.%, respectively.

### 2.2. Synthesis of Rh/TNTs and Rh/B–TNTs

The Rh/TNTs and Rh/B–TNTs were prepared by using the impregnation-photoreducing procedure.

A typical synthesis process is as follows: 1 g TNTs was added to 20 mL 2 wt.% RhAc2 aqueous solution, and then the suspension was vigorously agitated for 6 h. After low-energy sonication for 30 min, the suspension was centrifuged and the resulting solid was washed with a little deionized water to remove the ions adsorbed on the outer surface of TNTs, after which the solid was transferred into a 60 mL photo-reactor with 50 mL ethanol–water solution (*V*ethanol:*V*water = 9:1). The mixture was irradiated using a 300 W high-pressure mercury lamp, which ended with a color change of the mixture from blue to grey. Then, the mixture was centrifuged and the solid obtained was washed with deionized water and dried at 60 °C for 8 h in a vacuum. The catalyst obtained was labeled as Rh/TNTs (a_1_). The same procedures were used to prepare Rh/B–TNTs (b_1_), Rh/B–TNTs (c_1_) to just change TNTs to B–TNTs (b) and B–TNTs (c). The preparation process of Rh/B–TNTs (c_2_) and Rh/B–TNTs (c_3_) is similar to that of Rh/B–TNTs (c_1_), which had different Rh loadings (1 wt.% and 3 wt.% RhAc2).

### 2.3. Evaluation of Catalytic Performance of Catalysts for Hydroformylation

The catalytic activities of Rh/TNTs and Rh/B–TNTs for the hydroformylation of 2M3BN were evaluated. In a typical experiment, a 0.4 g catalyst and the required amount of substrate and solvent were placed in a 250-mL stainless steel autoclave reactor with a magnetic stirrer. The reactor was purged three times with H_2_ and then pressurized to 6.0 MPa with CO and H_2_ (CO/H_2_ = 1:1). After this, the reactor was heated to the desired temperature while stirring. When the reaction was over, the stirring was stopped. The reactor was then cooled to room temperature and the pressure was released gradually. The product was analyzed by GC7890B-5977A MS (Agilent, Santa Clara, CA, USA) or GC (GC-2014 gas chromatograph equipped with a 30 m × 0.53 mm SE-30 capillary column and a FID, Shimadzu, Japan,). Recycling uses of catalysts were carried out under the same conditions after recovering the catalyst from the reaction solution via centrifugation.

### 2.4. Characterization

The crystal phase and structure of catalysts were detected by XRD, Rigaku D/Max-2500, (Rigaku, Japan), which was performed with Cu Ka radiation (λ = 1.54 Å) at 2θ from 10° to 80°. TEM images were recorded using a Tecnai G2 F20 instrument (FEI, Hillsboro, OR, USA) at an accelerating voltage of 200 kV. The morphologies were analyzed by SEM, Shimadzu SS-550, Shimadzu, Japan,). The X-ray photoelectron spectrometer (XPS, Kratos Axis Ultra DLD multi-technique X-ray photoelectron spectra, Kratos Analytical Ltd., Manchester, UK) was used to test the chemical states of Rh in catalysts, and all binding energies were calibrated using C1s (*E*_b_ = 284.6 eV) for the reference. The contents of Rh and B were determined by ICP-AES (ICP-9000, USA Thermo Jarrell-Ash Corp, Franklin, MA, USA). N_2_ adsorption/desorption isotherms were collected on an Autosorb-1-MP 1530VP (Quantachrome, Florida, FL, USA) automatic surface area and porosity analyzer. The sample was degassed at 473 K for 5 h and then analyzed at 77 K. The relative pressure (*P*/*P*_0_) range used for the calculation of the BET surface area was from 0.05 to 0.30.

NH_3_-TPD measurement was performed on an Autosorb-IQ-C-XR automatic surface area and a porosity analyzer equipped with a thermal conductivity detector (TCD, Quantachrome, Florida, FL, USA). The samples were pretreated at 150 °C for 1 h in a flow of Ar, and then subjected to NH_3_ adsorption until saturation at room temperature. Desorbed NH_3_ was monitored at a heating rate of 10 °C/min from room temperature to 800 °C.

## 3. Results and Discussion

### 3.1. BET and ICP Analysis

A Brunauer–Emmett–Teller (BET) analysis was carried out to investigate the specific surface area (SSA) of the catalysts (Table 1). The actual contents of Rh and B in catalysts determined by the ICP were listed in Table 1. The SSA of TNTs (a), B–TNTs (b), and B–TNTs (c) were calculated to be 227.6, 238.6, and 275.8 m^2^/g, respectively. It can be observed that the BET surface area increased with the increasing content of B, an effect which can be attributed to the lower crystal size of the anatase phase in the B–TNTs supporter [28,29]. The SSA of all catalysts were significantly lower than that of pure supporters, which may be ascribed to the metal nanoparticles deposited on the outer and inner surface or which occupied the interspace between walls of TNTs [23,30,31,32].

### 3.2. XRD Analysis

**Figure 1:**
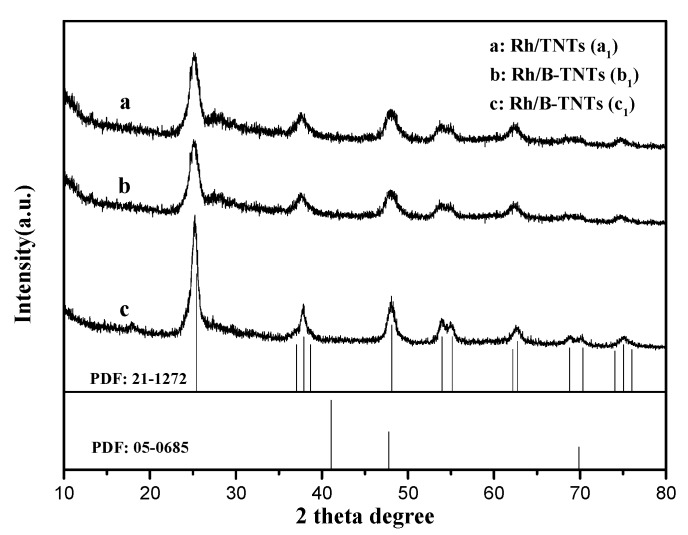
X-ray diffraction (XRD) patterns of Rh/TNTs and Rh/B–TNTs.

Figure 1 shows the XRD patterns of Rh/TNTs, Rh/B–TNTs. It can be seen from Figure 1 that all of the peaks can be perfectly indexed to anatase titania (PDF: 21-1272). The peaks at 2θ = 25.4°, 37.8°, 48.1°, 54.6°, 55.0°, 62.7°, 68.8°, and 75.0° can be assigned to diffractions of anatase TiO_2_ (101), (004), (200), (105), (211), (204), (116), and (215). There is no peak related to the Rh (PDF: 05-0685), which means that Rh particles are small and highly dispersed on the TNTs and B–TNTs, which is a similar result to that observed by previous reports [15,23].

### 3.3. SEM Analysis

**Figure 2:**
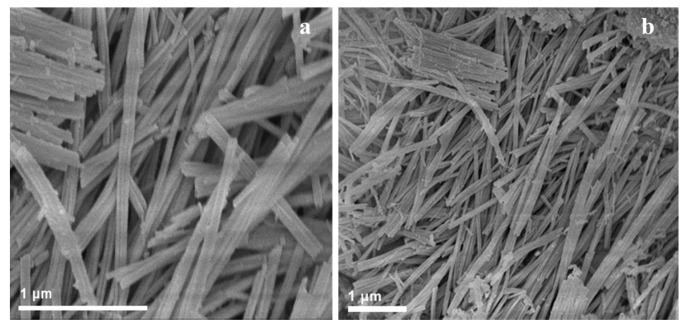
Scanning electron microscopy (SEM) images of a B–TNTs (**a**) and b B–TNTs (**b**).

Figure 2 shows the SEM images of the B–TNTs. As can be seen from Figure 2, the synthesized B–TNTs showed 1-D morphology with a length of up to hundreds of nanometers. We can see that the single nanotube in the B–TNTs samples is clearly discernible, showing a uniform diameter and a smooth surface.

### 3.4. TEM Analysis

**Figure 3:**
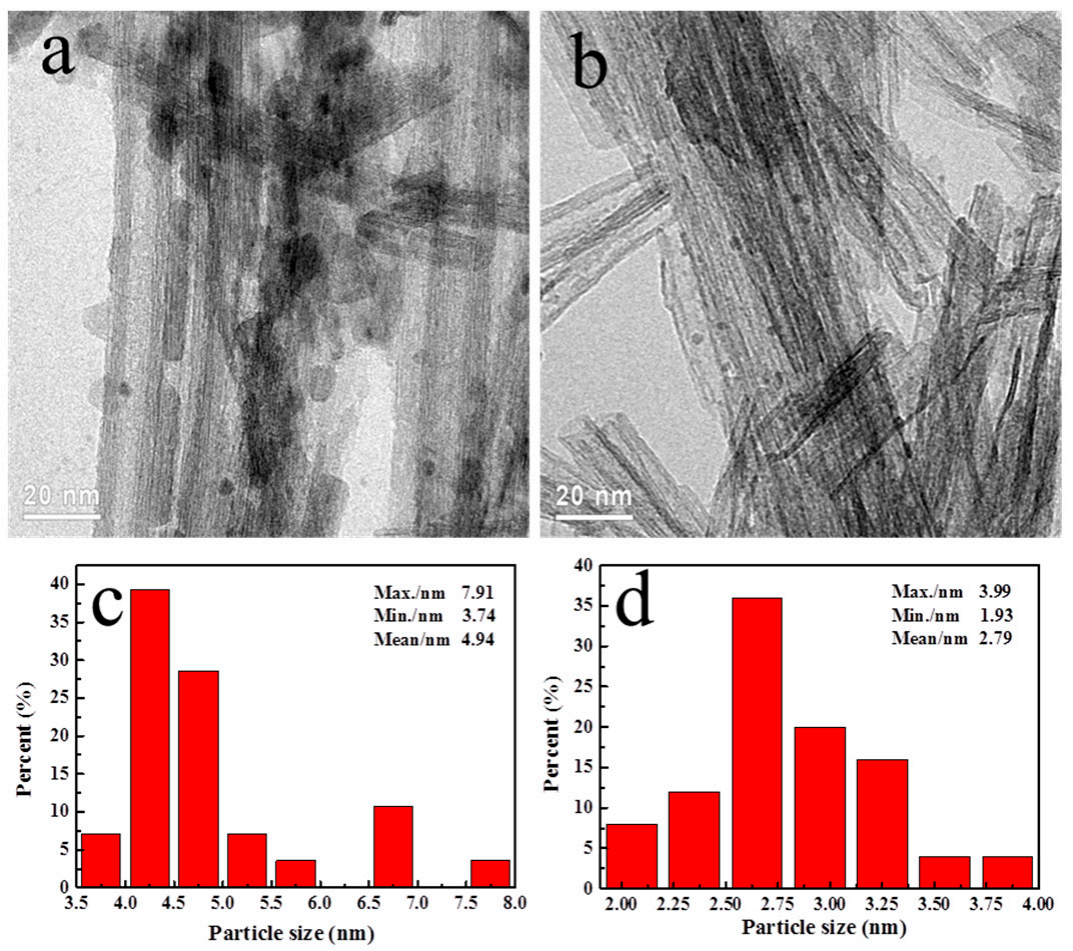
Transmission electron microscopy (TEM) images of (**a**) Rh/TNTs (a_1_); (**b**) Rh/B–TNTs (c_1_); Size distributions of particles in (**c**) Rh/TNTs (a_1_); (**d**) Rh/B–TNTs (c_1_).

Figure 3 shows the TEM images of the Rh/TNTs and Rh/B–TNTs. It is clear from Figure 3 that TNTs and B–TNTs show perfect nanotubular morphology with a length of about 200–300 nm and diameter of about 8–10 nm (Figure 3a,b). The nanotubes have a multiwalled structure with a spacing of about 7 nm. There is no obvious rupture or fracture caused by the light reduction process. These are attributable to the dispersed Rh-containing compound, which plays the same stable supporting role as Co and Fe compounds reported in the literature [16,27]. Rh nanoparticles are well-dispersed on the inner and outer surfaces of the nanotubes without agglomeration. Figure 3c,d show the size distributions of particles in different catalysts. The average diameter of the Rh particles in Rh/B–TNTs (c_1_) is about 2.79 nm (Figure 3d), which is obviously smaller than that of Rh/TNTs (a_1_) (4.94 nm, Figure 3c), and the size distributions of particles are in a relatively narrow range. These results mean that the presence of B in B–TNTs can effectively prevent the growth of Rh nanoparticles and improve the distributions of Rh particles, which is similar to the results found in literature [33]. 

### 3.5. XPS Analysis

**Figure 4:**
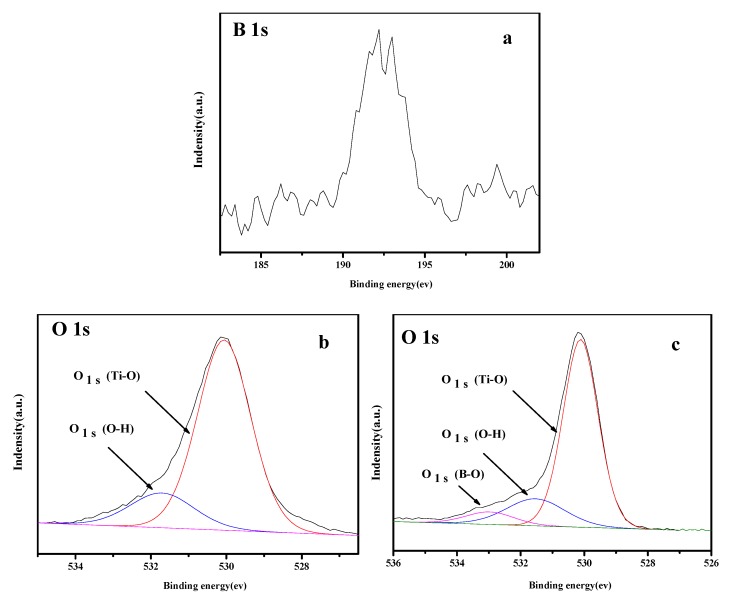

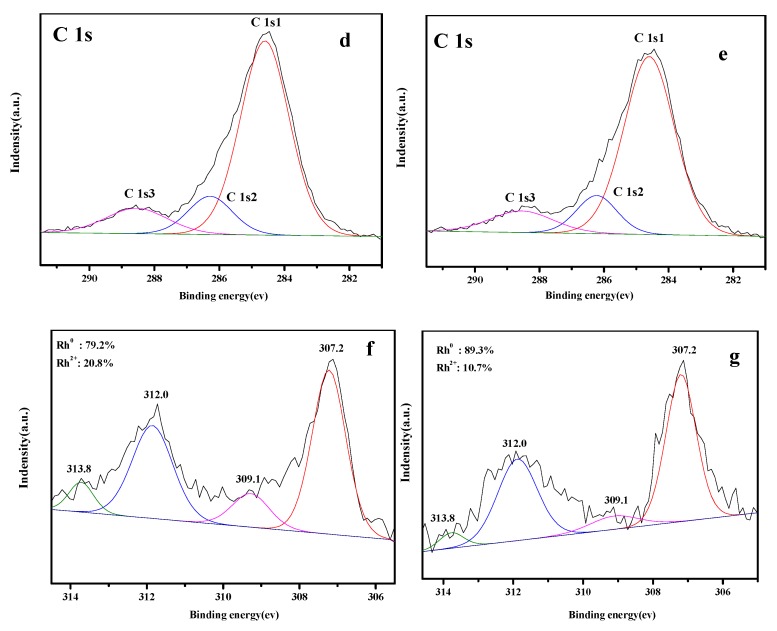
B 1s XPS spectrum of (**a**) Rh/B–TNTs (c_1_); O 1s XPS spectra of (**b**) Rh/TNTs (a_1_) and (**c**) Rh/B–TNTs (c_1_); C 1s XPS spectra of (**d**) Rh/TNTs (a_1_) and (**e**) Rh/B–TNTs (c_1_); XPS spectra of Rh in catalysts. (**f**) Rh/TNTs (a_1_), (**g**) Rh/B–TNTs (c_1_).

XPS was performed to evaluate the chemical state of the catalysts. As seen in Figure 4a, there was only one peak at about 192.4 eV for B 1s. As compared to the standard binding energy for B 1s in TiB_2_ (187.5 eV, Ti–B bonds) and in B_2_O_3_ (193.1 eV, B–O bonds), the binding energy of B 1s was between that of B_2_O_3_ and TiB_2_; thus, the boron atom was probably incorporated into TiO_2_ and the chemical environment surrounding the boron was likely to be Ti–B–O, which would be a similar result to those in previous reports [34,35].

Figure 4b,c display the O 1s spectra comparison between Rh/TNTs (a_1_) and Rh/B–TNTs (c_1_). From Figure 4b,c, the O 1s XPS spectra of the Rh/B–TNTs (c_1_) sample could be fitted by three peaks, corresponding to Ti–O (530.0 eV), the hydrogen group O–H (531.6 eV), and the B–O (533.0 eV) bond, respectively, while there were only two O 1s XPS peaks assigned to the Ti–O and O–H bonds, respectively of the Rh/TNTs (a_1_) sample, which also proved the presence of the B–O bond [35,36]. 

Figure 4d,e show that the carbon peaks consisted of three components. The peak at 284.6 eV corresponds to the binding energies of C 1s, and the second and third smaller peaks at 286.2 and 288.6 eV observed for both samples are probably due to impurity and air absorbents [37]. 

As shown in Figure 4f,g, Rh 3d_5/2_ and Rh 3d_3/2_ show that the group peaks not only centered at 307.2 and 312.0 eV, but also at 309.1 and 313.8 eV. These indicate that Rh exists in two forms of Rh^0^ and oxidation state. The higher binding energy state should have relevance to the incomplete reduction of Rh^2+^ ions; however, the electron transfers from Rh adatoms to the titanate substrate and the formation of partially oxidized states cannot be excluded, which is consistent with the literature [38]. It is well-known that Rh^0^ is the active site for the hydroformylation of olefins [15]. The percentage of Rh^0^ in rhodium is calculated based on the area of the fitted Gauss peaks. When the Rh loading is almost identical, the proportions of Rh^0^ in Rh/B–TNTs (c_1_) is 89.3%, which is higher than that in Rh/TNTs (a_1_). Thus, the incorporation of B can increase the proportion of Rh^0^ in the catalyst, promoting the hydroformylation of 2M3BN.

### 3.6. NH_3_-TPD Analysis

It has been reported that Lewis acid promoted CO insertion which favors hydroformylation over hydrogenation of alkenes [39]. Previous literature [40] reported that the incorporation of boron into Ti-substituted silicalite-2 zeolites (TS-2) could enhance both the number and strength of the acidic sites in TS-2.

**Figure 5:**
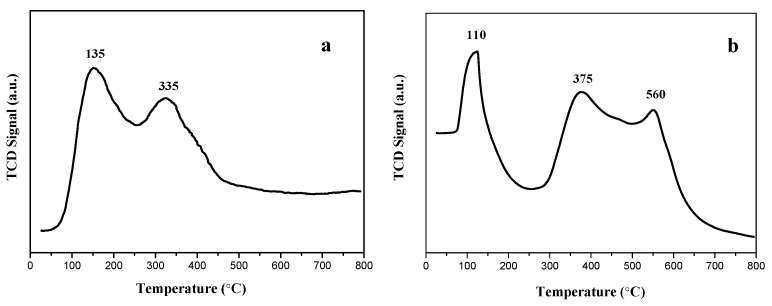
NH_3_-TPD plots of a TNTs (**a**) and (**b**) B–TNTs.

NH_3_-TPD was used to measure the acidic properties of TNTs (a) and B–TNTs (c), and the results are shown in Figure 5. There are two NH_3_ desorption peaks at 135 and 335 °C in the TNTs (Figure 5a), which are caused by the NH_3_ adsorbed on the acidic sites of the outer and inner surfaces of TNTs, respectively. It can be concluded that there are a lot of acidic sites on the inner and outer surfaces of TNTs, which is beneficial to the preferential adsorption of olefin to catalysts. Compared with TNTs (a), the B–TNTs (c) show three NH_3_ desorption peaks at around 110, 375, and 560 °C (Figure 5b), which is similar to the NH_3_-TPD results of Co-B/TNTs [41]. The peak at around 560 °C corresponding to NH_3_ adsorbed on stronger acidic sites implies that B may lead to the formation of a new kind of acidic site and enhance the acidity of catalysts.

### 3.7. Catalytic Activity Evaluation

**Scheme 1:**
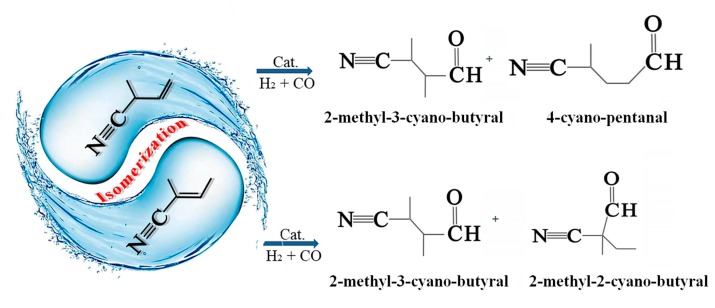
Possible aldehydes in the hydroformylation reaction of 2M3BN.

Scheme 1 shows the possible aldehydes formed in the hydroformylation reaction of 2M3BN.

In the real hydroformylation reaction of 2M3BN, an isomerization reaction of 2-methyl-2-butennitrile (2M2BN) may take place. Theoretically speaking, the products should include at least three kinds of aldehyde in the hydroformylation of 2M3BN, one of which is linear aldehyde and the others are branched-chain aldehydes (Figures S1–S3).

It is very interesting to study the selectivity for these aldehydes in the hydroformylation of 2M3BN and it can help us to understand how the –CN functional group affects the reaction process of the hydroformylation of olefin. The GC and MS fragments of products formed in the hydroformylation of 2M3BN are shown in the supporting information.

Table 2 shows the influence of B content on the activity of the catalyst. As can be seen from Table 2, the conversion rate of 2M3BN over all catalysts can reach up to 100%.

However, the selectivity for aldehyde in the reaction over Rh/B–TNTs is higher than that over Rh/TNTs. The selectivity for product aldehydes is shown to increase from 72% to 81%. In addition, the isomerization of 2M3BN clearly reduces from 25.8% to 17.9%. These results imply that in the catalytic processes, the presence of B in the catalysts may increase the rate of migratory insertion of the CO group, which suppresses the isomerization of 2M3BN and improves reaction selectivity for aldehydes. When the amount of B in Rh/B–TNTs increases from 0.56 wt.% to 0.99 wt.%, both of the selectivity for aldehyde and the l:b ratio of the product aldehydes increase. Thus, the suitable amount of B doping should be 0.99 wt.% in the present work.

Table 3 shows the effects of Rh content on the catalytic performance of the Rh/B–TNTs. It can be seen from Table 3 that the conversion rate of 2M3BN, the turnover frequency (TOF), and the selectivity for aldehydes all increase with an increase in Rh loadings from 0.09 wt.% to 0.16 wt.%. However, when the amount of Rh increases from 0.16 wt.% to 0.19 wt.%, the selectivity for aldehydes does not increase, but the ratio of linear aldehyde to the branched one clearly decreases. Thus, it can be concluded that the better rhodium content is 0.16wt.% for the catalyst Rh/B–TNTs.

Table 4 shows the effect of reaction temperature on the Rh/TNTs (a_1_) and Rh/B–TNTs (c_1_) catalyzed hydroformylation reaction of 2M3BN. Table 4 shows that the conversion of 2M3BN over Rh/TNTs, the TOF, and the ratio of l:b, all increased along with the increase in reaction temperature from 80 °C to 120 °C. For Rh/B–TNTs (c_1_), when the reaction temperature increased from 80 °C to 120 °C, the conversion of 2M3BN, the total amount of aldehydes, and the ratio of l:b, all improved. Thus, it can be concluded that the suitable reaction temperature should be 120 °C. The difference is that under the almost identical Rh loading and reaction temperature, the activity of Rh/B–TNTs is significantly higher than that of Rh/TNTs. This clearly demonstrates that the presence of B in the catalysts is greatly beneficial for the hydroformylation reaction of 2M3BN. However, the isomerization of 2M3BN increases when the temperature increases from 80 °C to 120 °C, indicating that increasing the temperature is more favorable for the isomerization of 2M3BN.

The stability of catalysts is important for the hydroformylation of olefins for practical application. It was reported that the stability of Ni catalysts can be enhanced by boron doping [42]. Density functional theory calculations also suggested that the presence of boron could improve the stability of the catalyst by selectively blocking the deposition, nucleation, and growth of resilient carbon species of Co catalysts under Fischer-Tropsch synthesis conditions [43].

To investigate the stability of the catalysts, Rh/TNTs (a_1_) and Rh/B–TNTs (c_1_) were selected for recycle catalytic experiments. The experimental results are listed in Table 5. As can be seen from this Table, Rh/B–TNTs (c_1_) remained highly active and the yield of aldehyde maintained at around 67.8% in the fourth recycles. However, the Rh/TNTs (a_1_) shows poor stability. We used ICP to test the rhodium content in the solution after the reaction (listed in Table 6). We can clearly see that the addition of B can significantly reduce the loss of rhodium. The Rh content is about 18.8 PPM in the solution after the first reaction used for the Rh/B–TNTs(c_1_) catalyst and is much smaller than that in the solution used for the Rh/TNTs(a_1_) catalyst, which is about 42.8 PPM. The experimental results show that boron doping can greatly improve the catalytic stability of the catalyst.

## 4. Conclusions

This study showed that the high catalytic activity of B-modified Rh/TNTs catalysts for olefin hydroformylation could be improved by increasing the acid site of the catalyst. The Rh/B–TNTs catalysts not only display good catalytic performance, but also good circulation. The catalysts could be easily recovered and were used up to four times. Although we observed a modest decrease of activity with the recycling of the catalyst, the selectivity decreased quite a bit from 81% to 68%, and Rh leaching was also observed. However, the stability of the catalyst Rh/B–TNTs(c_1_) was much better than that of Rh/TNTs(a_1_). In conclusion, the introduction of B can not only improve the catalytic activity of the catalyst, but also improve its stability. 

## Figures and Tables

**Table 1:** Brunauer–Emmett–Teller (BET) and atomic emission spectrometer (ICP) data of TNTs, Rh/TNTs, B–TNTs and Rh/B–TNTs.

Entry	Catalyst	SSA (m^2^/g)	Rh Content (wt.%)	B Content (wt.%)
1	TNTs(a)	227.6	-	-
2	Rh/TNTs(a_1_)	198.6	0.13	-
3	B–TNTs(b)	238.6	-	0.56
4	Rh/B–TNTs(b_1_)	225.2	0.16	0.56
5	B–TNTs (c)	275.8	0	0.99
6	Rh/B–TNTs(c_1_)	268.6	0.16	0.99
7	Rh/B–TNTs(c_2_)	270.2	0.09	0.99
8	Rh/B–TNTs(c_3_)	267.4	0.19	0.99

**Table 2:** Effect of boron content on the hydroformylation over Rh/B–TNTs ^a^.

Catalyst	Content of B (wt.%)	Conversion (%)	Isomerism ^d^ (%)	TOF ^b^ (h^−1^)	Aldehyde (%)	b:l ^c^
Rh/TNTs(a_1_)	0	100	25.8	2663	72	32:68
Rh/BTNTs(b_1_)	0.56	100	20.4	2532	79	33:67
Rh/BTNTs(c_1_)	0.99	100	17.9	3576	81	31:69

^a^ Reaction conditions: 2M3BN = 5 mL, catalyst = 0.40 g, temp.= 120 °C, syngas pressure = 6.0 MPa, CO:H_2_ = 1, solvent (toluene) = 70 mL, and reaction time = 2 h. ^b^ TOF is calculated by the formula $\mathrm{TOF}=\frac{n\left(\mathrm{aldehydes}\right)}{n\left(\mathrm{Rh}\right)\times h}$, *n*(aldehydes) is the total moles of aldehydes, *n*(Rh) is the total moles of Rh in the catalyst. ^c^ b:l is branched aldehyde: linear aldehyde. ^d^ $\mathrm{Isomerism}=\frac{n\left(2-\mathrm{methyl}-2-\mathrm{cyano}-\mathrm{butyral}\right)+n\left(2M2\mathrm{BN}\right)}{n\left(2M3\mathrm{BN}\right)}$, *n*(2M2BN) is the amount of 2M2BN in the liquid after reaction, *n*(2M3BN) is the amount of reaction substrate.

**Table 3:** Effect of Rh loading in Rh/B–TNTs on the hydroformylation of 2M3BN ^a^.

Catalyst	Rh Content (wt.%)	Conversion (%)	Isomerism ^d^ (%)	TOF ^b^ (h-1)	Aldehyde (%)	b:l ^c^
Rh/B–TNTs(c_1_)	0.16 (6.2 × 10^−3^ mmol)	100	17.9	3576	81	31:69
Rh/B–TNTs(c_2_)	0.09 (3.5 × 10^−3^ mmol)	86	19.1	2742	68	32:68
Rh/B–TNTs(c_3_)	0.19 (7.3 × 10^−3^ mmol)	100	18.2	3639	79	42:58

^a^ Reaction conditions: 2M3BN = 5 mL, catalyst = 0.40 g, temp.= 120 °C, syngas pressure = 6.0 MPa, CO:H_2_ = 1, solvent (toluene) = 70 mL, and reaction time = 2 h. ^b^ TOF is calculated by the formula $\mathrm{TOF}=\frac{n(\mathrm{aldehydes})}{n\left(\mathrm{Rh}\right)\times h}$, *n*(aldehydes) is the total moles of aldehydes, *n*(Rh) is the total moles of Rh in catalyst. ^c^ b:l is branched aldehyde: linear aldehyde. ^d^ $\mathrm{Isomerism}=\frac{n\left(2-\mathrm{methyl}-2-\mathrm{cyano}-\mathrm{butyral}\right)+n(2M2\mathrm{BN})}{n(2M3\mathrm{BN})}$, *n*(2M2BN) is the amount of 2M2BN in the liquid after reaction, *n*(2M3BN) is the amount of reaction substrate.

**Table 4:** Effect of reaction temperatures on the Rh/TNTs- and Rh/B–TNTs-catalyzed hydroformylation reaction of 2M3BN ^a^.

Catalyst	Temperature (°C)	Conversion (%)	Isomerism ^d^ (%)	TOF ^b^ (h^−1^)	Aldehyde (%)	b:l ^c^
Rh/TNTs (a_1_)	80	66	19.5	1833	46	65:35
Rh/TNTs (a_1_)	100	100	20.4	3152	73	39:61
Rh/TNTs (a_1_)	120	100	25.9	3263	72	32:68
Rh/B–TNTs (c_1_)	80	74	13.1	1892	59	62:38
Rh/B–TNTs (c_1_)	100	100	16.8	3537	81	41:59
Rh/B–TNTs (c_1_)	120	100	17.9	3576	81	31:69

^a^ Reaction conditions: 2M3BN = 5 mL, Catalyst = 0.40 g, syngas pressure = 6.0 MPa, CO:H_2_ = 1, solvent (toluene) = 70 mL, and reaction time = 2 h. ^b^ TOF is calculated by the formula $\mathrm{TOF}=\frac{n(\mathrm{aldehydes})}{n\left(\mathrm{Rh}\right)\times h}$, *n*(aldehydes) is the total moles of aldehydes, *n*(Rh) is the total moles of Rh in catalyst. ^c^ b:l is branched aldehydee: linear aldehyd. ^d^ $\mathrm{Isomerism}=\frac{n\left(2-\mathrm{methyl}-2-\mathrm{cyano}-\mathrm{butyral}\right)+n(2M2\mathrm{BN})}{n(2M3\mathrm{BN})}$, *n*(2M2BN) is the amount of 2M2BN in the liquid after reaction, *n*(2M3BN) is the amount of reaction substrate.

**Table 5:** Studies of catalyst stability ^a^.

Catalyst	Cycle Times	Conversion (%)	Isomerism ^d^ (%)	TOF ^b^ (h^−^^1^)	Aldehyde (%)	b:l ^c^
Rh/B–TNTs(c_1_)	1	100	17.9	3576	81	31:69
Rh/B–TNTs(c_1_)	2	100	20.3	3312	77	29:71
Rh/B–TNTs(c_1_)	3	92	18.8	2556	80	31:69
Rh/B–TNTs(c_1_)	4	90	29.1	2202	68	38:62
Rh/TNTs(a_1_)	1	100	25.9	3263	72	32:68
Rh/TNTs(a_1_)	2	50	28.6	1665	69	35:65
Rh/TNTs(a_1_)	3	trace	30.5	-	trace	-

^a^ Reaction conditions: 2M3BN = 5 mL, Rh/TNTs (a_1_) = 0.40 g, Rh/B–TNTs (c_1_) = 0.40 g, temp. = 120 °C, syngas pressure = 6.0 MPa, CO:H_2_ = 1, solvent (toluene) = 70 mL. ^b^ TOF is calculated by the formula $\mathrm{TOF}=\frac{n\left(\mathrm{aldehydes}\right)}{n\left(\mathrm{Rh}\right)\times h}$, *n*(aldehydes) is the total moles of aldehydes, *n*(Rh) is the total moles of Rh in catalyst. ^c^ b:l is branched aldehyde: linear aldehyde. ^d^ $\mathrm{Isomerism}=\frac{n\left(2-\mathrm{methyl}-2-\mathrm{cyano}-\mathrm{butyral}\right)+n\left(2M2\mathrm{BN}\right)}{n\left(2M3\mathrm{BN}\right)}$, *n*(2M2BN) is the amount of 2M2BN in the liquid after reaction, *n*(2M3BN) is the amount of reaction substrate.

**Table 6:** Rhodium content in the solution after the reaction.

Catalyst	Cycle Times	Rh (PPM)
Rh/B–TNTs(c_1_)	1	18.8
Rh/B–TNTs(c_1_)	2	15.7
Rh/B–TNTs(c_1_)	3	13.0
Rh/B–TNTs(c_1_)	4	11.2
Rh/TNTs(a_1_)	1	42.8
Rh/TNTs(a_1_)	2	28.7
Rh/TNTs(a_1_)	3	8.6

## Supplementary Materials

The following are available online at http://www.mdpi.com/2079-4991/8/10/755/s1, Figure S1: Gas chromatogram of hydroformylation. **a** before the reaction; **b** after the reaction. Figure S2: GC-Mass profiles of samples from hydroformylation of 2M3BN in toluene. Fragment ions of the 2-methyl-2-cyano-butyral (RT = 9.47 min); b fragment ions of the 2-methyl-3-cyano-butyral (RT = 10.30 min); c fragment ions of the 4-cyano-pentanal (RT = 13.70 min). Figure S3: IR spectra of hydroformylation production of 2M3BN.
