# Supplementary material for: Synthesis and Characterization of Rh/B–TNTs as a Recyclable Catalyst for Hydroformylation of Olefin Containing –CN Functional Group
Source: Nanomaterials (Basel). 2018 Sep 25;8(10):755. doi: 10.3390/nano8100755 (PMC6215287; doi:10.3390/nano8100755)
Supplement: Supplementary file 1 [file nanomaterials-08-00755-s001.pdf]

Synthesis and characterization of Rh/B-TNTs as a reusable catalyst for hydroformylation of olefin containing -CN functional group

Penghe Su [1], Xiaotong Liu [1], Ya Chen [1], Hongchi Liu[1], Baolin Zhu [1,2], Shoumin Zhang[1,2], Weiping Huang [1,2,3*]

1 College of Chemistry, Nankai University, Tianjin 300071, China;

2 The Key Laboratory of Advanced Energy Materials Chemistry (Ministry of Education), Nankai University, Tianjin 300071, China;

3 Collaborative Innovation Center of Chemical Science and Engineering, Tianjin 300071, China;

Supporting Information

Correspondence: hwp914@nankai.edu.cn

Tel.: +86-138-2009-6974

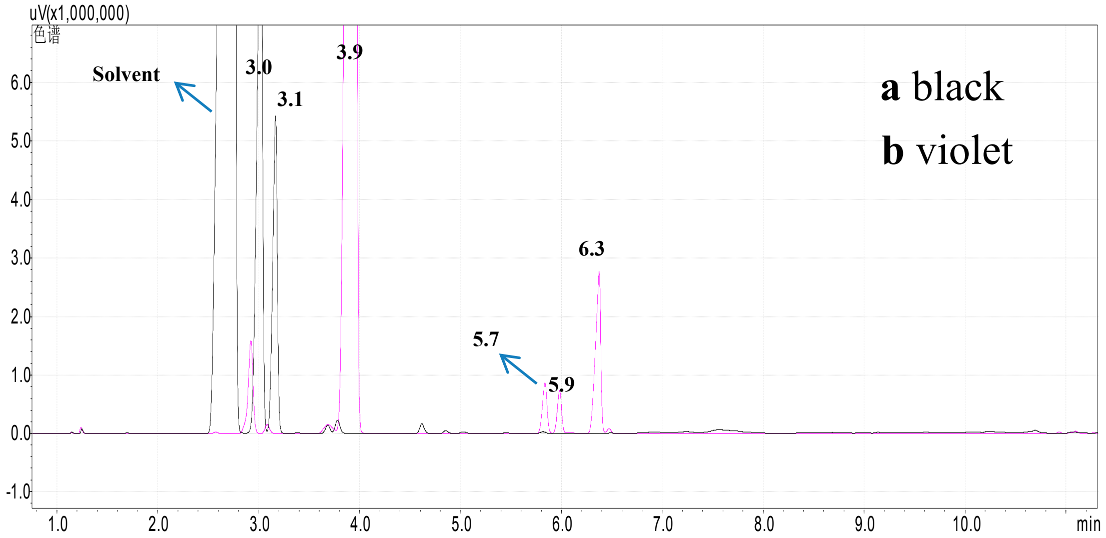

2M3BN (RT: 3.0min); 2M2BN (RT: 3.1 min); toluene (RT: 3.9 min); 2-methyl-2-cyano-butyral (RT: 5.7 min); 2-methyl-3-cyano-butyral (RT: 5.9 min); 4-cyano-pentanal (RT: 6.3 min).

Figure S1. Gas chromatogram of hydroformylation: **(a)** before the reaction; **(b)** after the reaction.

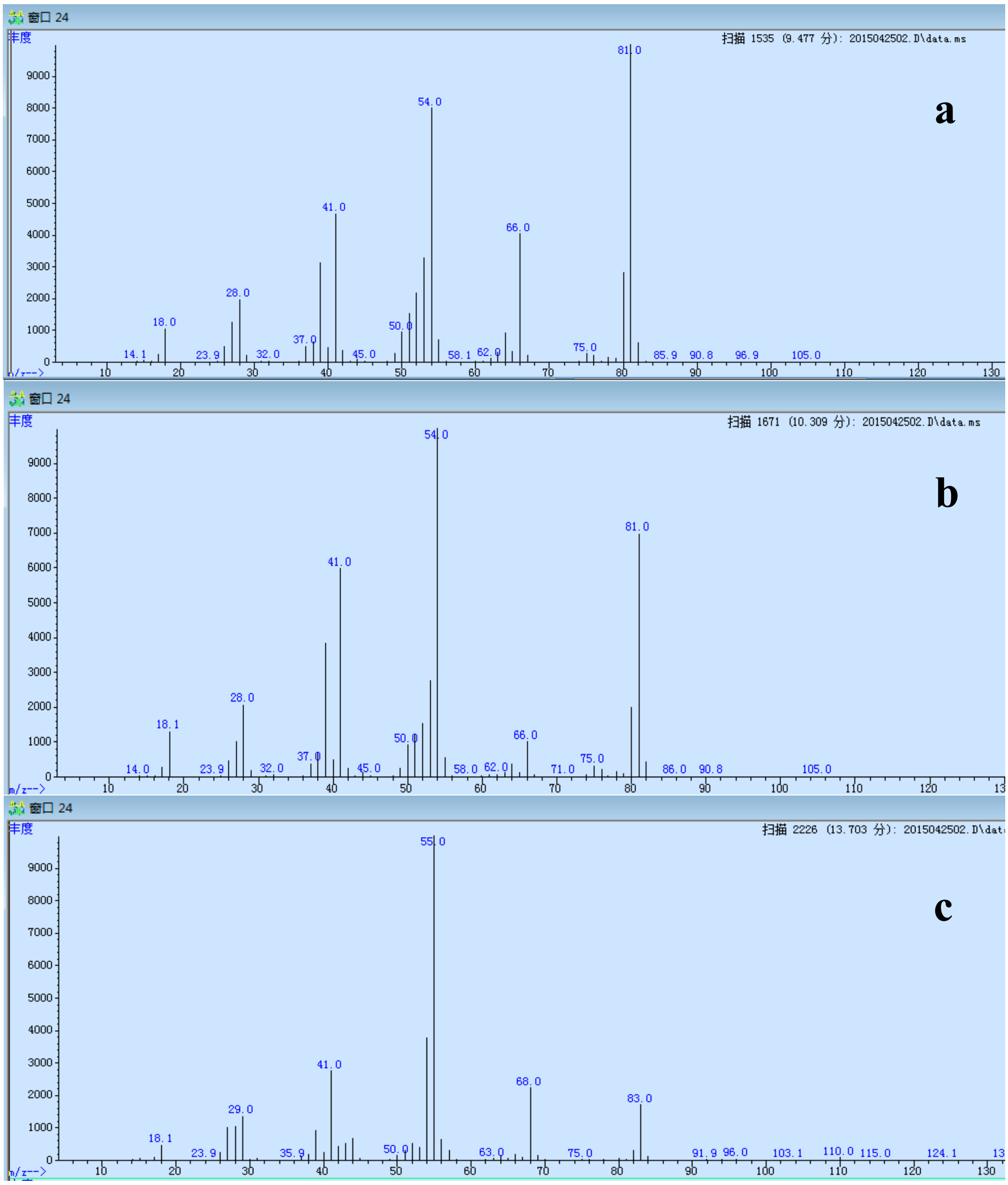

Figure S2. GC-Mass profiles of samples from the hydroformylation of 2M3BN in toluene. **(a)** Fragment ions of the 2-methyl-2-cyano-butyral (RT = 9.47 min); **(b)** fragment ions of the 2-methyl-3-cyano-butyral (RT = 10.30 min); **(c)** fragment ions of the 4-cyano-pentanal (RT = 13.70 min).

Figure S2 shows the GC-Mass profiles of the sample from the hydroformylation of 2M3BN in toluene. Although the fragmentation peaks of aldehyde products can be seen in the mass spectrometry, no obvious molecular ion peaks can be seen because of the easy abscission of aldehydes. In order to further confirm the presence of aldehyde groups in the products, the products of the hydroformylation were analyzed with IR, and the results are shown in Figure S3.

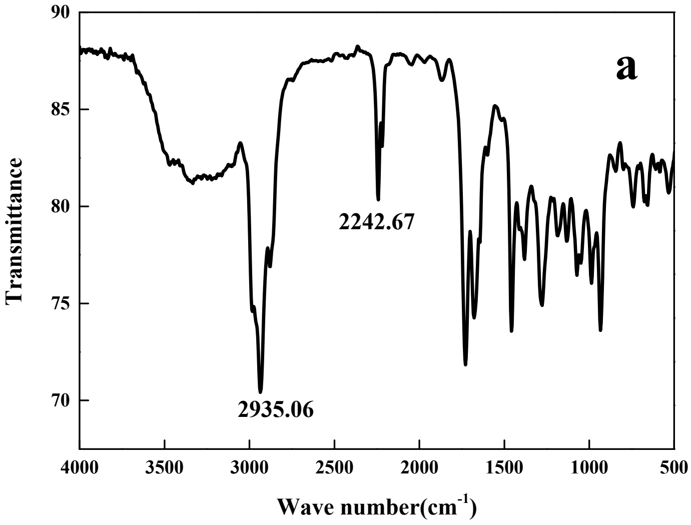

Figure S3. IR spectra of hydroformylation production of 2M3BN.

As shown in Figure S3, the characteristic peaks at 2242 cm^{-1} and 2935 cm^{-1} belong to the cyano group and aldehyde group, respectively. It can be seen that the aldehyde product is indeed present in the hydroformylation product of 2M3BN.
